# Interferometric modulation for generating extended light sheet: improving field of view

**DOI:** 10.1117/1.JBO.29.4.046501

**Published:** 2024-04-16

**Authors:** Jixiang Wang, Xin Xu, Hong Ye, Xin Zhang, Guohua Shi

**Affiliations:** a University of Science and Technology of China, School of Biomedical Engineering (Suzhou), Division of Life Sciences and Medicine, Hefei, China; b Chinese Academy of Science, Suzhou Institute of Biomedical Engineering and Technology, Jiangsu Key Laboratory of Medical Optics, Suzhou, China

**Keywords:** light sheet microscopy, interference, field of view, axial resolution

## Abstract

**Significance:**

Light-sheet fluorescence microscopy (LSFM) has emerged as a powerful and versatile imaging technique renowned for its remarkable features, including high-speed 3D tomography, minimal photobleaching, and low phototoxicity. The interference light-sheet fluorescence microscope, with its larger field of view (FOV) and more uniform axial resolution, possesses significant potential for a wide range of applications in biology and medicine.

**Aim:**

The aim of this study is to investigate the interference behavior among multiple light sheets (LSs) in LSFM and optimize the FOV and resolution of the light-sheet fluorescence microscope.

**Approach:**

We conducted a detailed investigation of the interference effects among LSs through theoretical derivation and numerical simulations, aiming to find optimal parameters. Subsequently, we constructed a customized system of multi-LSFM that incorporates both interference light sheets (ILS) and noninterference light-sheet configurations. We performed beam imaging and microsphere imaging tests to evaluate the FOV and axial resolution of these systems.

**Results:**

Using our custom-designed light-sheet fluorescence microscope, we captured the intensity distribution profiles of both interference and noninterference light sheets (NILS). Additionally, we conducted imaging tests on microspheres to assess their imaging outcomes. The ILS not only exhibits a larger FOV compared to the NILS but also demonstrates a more uniform axial resolution.

**Conclusions:**

By effectively modulating the interference among multiple LSs, it is possible to optimize the intensity distribution of the LSs, expand the FOV, and achieve a more uniform axial resolution.

## 1 Introduction

Light-sheet fluorescence microscopy (LSFM) has emerged as a powerful and versatile imaging technique renowned for its remarkable features, including high-speed 3D tomography, minimal photobleaching, and low phototoxicity. This imaging modality has garnered widespread usage across diverse disciplines, encompassing brain neuroscience,^1^^–^^3^ embryonic development,^4^^,^^5^ cell biology,^6^ organoid morphology,^7^ and among other areas of research. Notably, LSFM has witnessed substantial progress and innovation over the past decade, propelling its rapid development and facilitating its wide adoption.

In LSFM, there are two typical types of light sheets (LSs): static planar LS is generated by a cylindrical lens,^8^ and virtual LS is formed by a beam scanner.^9^ In the field of LSFM, two crucial parameters are the field of view (FOV) size and axial resolution. However, there exists a trade-off between these two parameters, namely, the challenge of expanding the FOV while simultaneously maintaining a high axial resolution. Traditional LSFM typically employs Gaussian beams for illumination.^8^^–^^10^ Due to the proportional relationship between the FOV size and the square of the axial resolution of Gaussian LS (GLS),^11^ expanding the FOV unavoidably leads to a compromise in axial resolution. Furthermore, GLS fluorescence microscopy exhibits nonuniform axial resolution, with higher axial resolution at the center of the FOV and lower axial resolution at the edges. Maintaining high resolution and uniform resolution while expanding the FOV is a significant challenge. To address this challenge, researchers have utilized the properties of nondiffracting beams, which have the characteristic of maintaining a constant beam width regardless of propagation distance.^12^ They have developed nondiffracting LSs, such as Airy LSFM,^13^^–^^15^ Bessel LSFM,^16^^–^^18^ and lattice LSFM,^19^^,^^20^ to expand the FOV while preserving the uniformity of axial resolution. However, as the FOV increases, the side lobes of nondiffracting beams also increase, leading to a decrease in tomographic capabilities and an increased risk of out-of-focus fluorescence bleaching.^21^ Additionally, some new techniques, such as tiled LSs^22^^,^^23^ and axial scanning LSs,^24^^–^^26^ can expand the FOV while maintaining uniform axial resolution. However, these methods require additional camera exposure time, sacrificing imaging speed. In recent years, the concept of multiple LS illuminations has been proposed to expand the FOV in LSFM without sacrificing camera exposure time,^27^^,^^28^ thereby maintaining LSFM imaging speed. However, due to inevitable mutual interference between multiple LS, researchers have implemented measures such as laterally separating the beams and subtracting complementary images^28^ or precisely designing the optical path length of each beam in the optical system to exceed the coherence length of the light source.^27^ These measures are taken to avoid interference between LSs. However, it is important to note that these additional measures increase the complexity of the system.

Based on the relative lack of research on the interference effects between multiple LSs, our aim is to further explore and improve this field. The primary objective of this study is to investigate the interference effects between LSs and discover how interference behavior can positively contribute to achieving larger FOV and more uniform axial resolution in LSFM. We refer to these LS as interference light sheets (ILS). By conducting theoretical analyses of the interference between two LSs and identifying specific interference parameters, we performed numerical simulations and compared it with noninterference light sheets (NILS) and GLS. The results indicate that the FOV of the ILS is ∼3 times that of GLS, and the NILS only achieve about 1.56 times the FOV of GLS. Moreover, the ILS demonstrates a more uniform axial resolution. To validate the performance of the ILS fluorescence microscope (ILSFM), we constructed an experimental system integrating different types of LSs and conducted verification using fluorescence bead imaging.

## 2 Theory

**Fig. 1:**
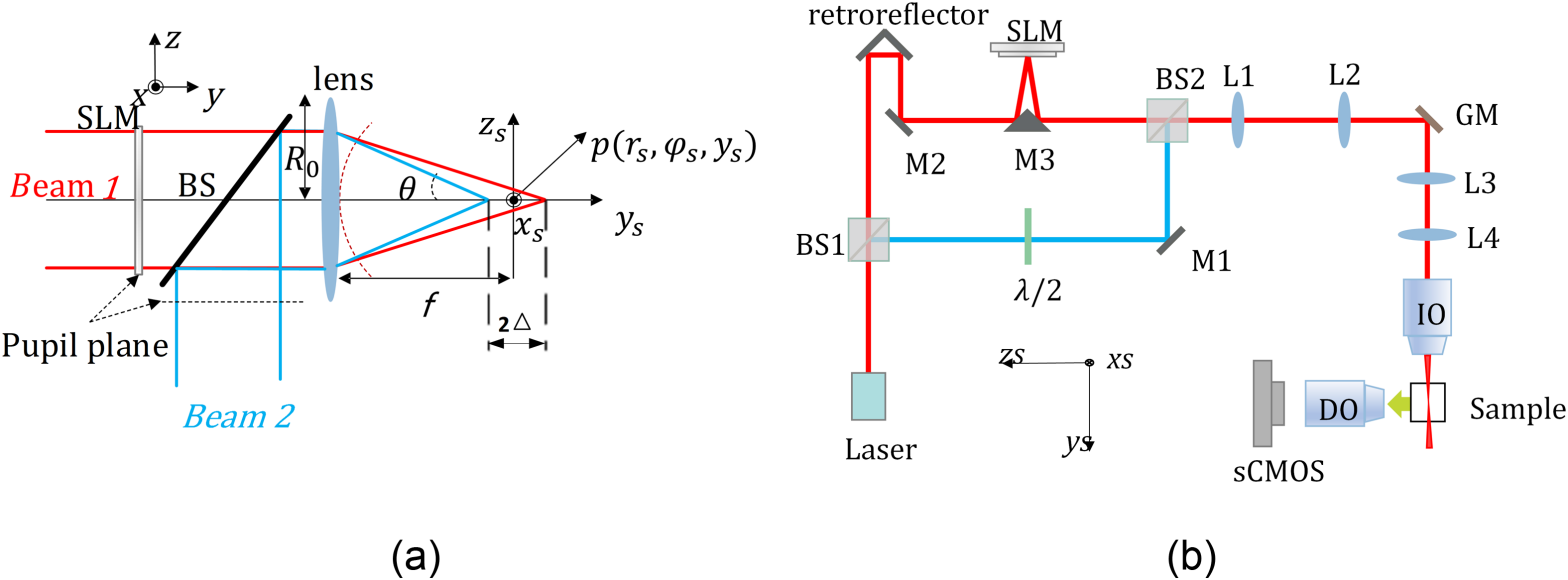
Generation of the ILS. (a) Principle of interference of two LS separated by a distance 2Δ. SLM is located at the conjugated pupil plane of the objective lens to modulate the phase of the beam $E_{1}$. BS is used to couple the beams. The objective pupil radius is $R_{0}$, and the focal length is f. The pupil plane coordinates are x, y, and z; the objective lens focus space coordinates $x_{s}$, $y_{s}$, and $z_{s}$; $p(r_{s},\varphi_{s},y_{s})$ point is located near the focus. (b) Schematic diagram of LSFM system. GM, galvanometer mirror; BS, beam splitter; L, lens; M, mirror; SLM, spatial light modulator; IO, illuminated objectives; DO, detection objectives; and sCMOS, camera.

As is well known, a virtual LS is formed by scanning a focused beam of light along one dimension. In the coordinate system depicted in Fig. 1(a), the focused beam of light coming from the objective lens can be mathematically described using the Born–Wolf formula:^29^

$$E_{\mathrm{ill}}(r_{s},\theta_{s},\varphi_{s})=-\frac{ik}{2\pi }\int_{0}^{\beta }\int_{0}^{2\pi }P(r)*E_{\mathrm{in}}(r)\exp[i\Psi (r_{s},\theta_{s},\varphi_{s},\varphi ,\theta )]\sin(\theta )d\varphi \;d\theta ,$$ (1a)

$$\Psi (r_{s},\theta_{s},\varphi_{s},\varphi ,\theta )=(k_{0}\Phi_{s})+(kr_{s}\;\sin(\theta_{s})\sin(\theta )\cos(\varphi -\varphi_{s}))+(ky_{s}\;\cos(\theta )),$$ (1b)

where $E_{\text{ill}}$ $\left(r_{s},\theta_{s},\varphi_{s}\right)$ represents the electric field vector at a given point, defined in cylindrical coordinates as shown in Fig. 1(a). $\Phi_{s}$ represents the initial aberration function, k denotes the wave vector, θ represents the apex angle, and φ represents the azimuth angle. NA=n*sin(β), where n represents the refractive index of the medium, and NA is the numerical aperture. P(r) is the pupil entry function, and $E_{\text{in}}$ (r) denotes the electric field intensity after the incident light passes through the objective lens. If the incident beam is a Gaussian beam and P(r)=1, under ideal conditions, the intensity of light near the focal point of the objective lens can be simply approximated as a Gaussian beam:

$$I(r_{s},y_{s})=\text{Eill}*{\text{Eill}}^{*}=I_{0}*\frac{w_{0}^{2}}{w^{2}(y_{s})}\;\exp(-\frac{2r_{s}^{2}}{w^{2}(y_{s})}),$$ (2)

where $w_{0}$ is the waist radius, $I_{0}$ is the intensity at the center of the beam waist, and $w(y_{s})=w_{0}[1+(y_{s}/Y_{r}{)}^{2}{]}^{\left(1/2\right)}$ is the beam radius at $y_{s}$, The FOV of the GLS is approximately twice the Rayleigh length, $\mathrm{FOV}=2Y_{r}$, $Y_{r}=\pi w_{0}^{2}/\lambda$ is Rayleigh length, and λ is the wavelength.

For a coherent light source, by employing a beam splitter (BS) and a spatial light modulator (SLM) loaded with defocused phase, two beams can be generated at the focal plane behind the objective lens, separated by a distance of 2Δ, as depicted in Fig. 1(a). Assuming the polarization of both beams is aligned along the x axis, and considering that the focal points are located at Δ and −Δ, respectively, the resulting intensity of the double-beam interference can be expressed as

$$I_{\text{interference}}=I(r_{s},y_{s}-\Delta )+I(r_{s},y_{s}+\Delta )+2\sqrt{I(r_{s},y_{s}-\Delta )I(r_{s},y_{s}+\Delta )}\;\cos(\Phi (r_{s},y_{s})),$$ (3)

where $I(r_{s},y_{s}-\Delta )$ and $I(r_{s},y_{s}+\Delta )$ represent the intensity of the two beams, respectively, and Φ represents the phase difference between the two beams. For the Gaussian beam, the phase difference Φ can be expressed as^30^

$$\Phi (r_{s},y_{s})=\frac{kr_{s}^{2}}{2}[\frac{1}{\varsigma (y_{s}+\Delta )}-\frac{1}{\varsigma (y_{s}-\Delta )}]+[\arctan(\frac{y_{s}-\Delta }{Y_{r}})-\arctan(\frac{y_{s}+\Delta }{Y_{r}})]+(2k\Delta +\delta ),$$ (4)

where ζ represents the curvature radius of the wavefront of optical waves, $\zeta (y_{s}+\Delta )=(y_{s}+\Delta )*{\left\{1+{\left[Y_{r}/(y_{s}+\Delta )\right]}^{2}\right\}}^{1/2}$, δ represents the initial phase difference, with 0<δ<2π, it can be adjusted using an SLM, and for $y_{s}=0$, we set $\alpha =\Delta /Y_{r}$. Then Eq. (4) is simplified to Eq. (5)

$$\Phi (r_{s},y_{s}=0)=\frac{r_{s}^{2}}{w_{0}^{2}}*\frac{\alpha }{1+\alpha^{2}}+\Phi_{0},$$ (5)

$$\Phi_{0}=2k\Delta +\delta -2\;\arctan(\alpha ).$$ (6)

By substituting Eqs. (5) and (6) into Eq. (3), the interference light intensity in the $y_{s}=0$ plane is

$$I_{\text{interference}}(r_{s},y_{s}=0)=4I_{\Delta }\;\cos^{2}[\frac{r_{s}^{2}}{w_{0}^{2}}*\frac{\alpha }{1+\alpha^{2}}+\frac{\Phi_{0}}{2}],$$ (7)

where

$$I_{\Delta }=I(r_{s},0-\Delta )=I(r_{s},\Delta )=I_{0}*\frac{1}{1+\alpha^{2}}\;\exp(-\frac{2r_{s}^{2}}{(1+\alpha^{2})w_{0}^{2}}).$$ (8)

When $r_{s}=0$, the intensity along the propagation axis can be expressed as

$$I(r_{s}=0,y_{s})=I_{0}w_{0}^{2}\{\frac{1}{w^{2}(y_{s}-\Delta )}+\frac{1}{w^{2}(y_{s}+\Delta )}+\frac{2\;\cos[\Phi (r_{s}=0,y_{s})]}{w(y_{s}-\Delta )w(y_{s}+\Delta )}\}.$$ (9)

For an interfering beam, the intensity is modulated by the square of the cosine function. From Eq. (7), we can determine the first zero point of the interference beam

$$r_{s0}=w_{0}\sqrt{\left(\frac{(2m+1)\pi }{2}-B)(\frac{1+\alpha^{2}}{\alpha }\right)},$$ (10a)

$$B=\mathrm{mod}(\Phi_{0}/2,\pi ),$$ (10b)

m=[sign(B−π/2)+1]/2, (10c)

where sign(.) represents the sign function, it can be observed from Eqs. (7) and (10) that by choosing appropriate parameters, a smaller beam size can be achieved. For example, when $w_{0}=2.2\;\mu m$, α=2, and 2.07<δ<6.06, the value of $r_{s0}$ is smaller than w. Furthermore, it is worth noting that there are solutions where $r_{s0}$ is smaller than $w_{0}$. For instance, when $w_{0}=2.2\;\mu m$, α=0.6, and δ=0.14, the ratio $r_{s0}/w_{0}$ is 0.77, it means that the focal point is reduced by a factor of 1.3. This situation may contribute to the generation of optical spots beyond the diffraction limit. However, as it is beyond the scope of this paper, it will not be discussed in detail.

For noninterfering beams, the intensity can be simply represented as the superposition of intensities

$$I_{\text{noninterference}}=I(r_{s},y_{s}-\Delta )+I(r_{s},y_{s}+\Delta ).$$ (11)

Clearly, at a distance Δ from the waist of the minimum beam, the width of the noninterfering spot is the same as that of the Gaussian beam. However, each beam, at the focal point of the other beam, exhibits diverging light, resulting in an apparent enlargement of the focal spot. This diffusion of light is undesirable for LS fluorescence imaging of specimens since it can excite background fluorescence.

## 3 Simulation Results

In the preceding analysis and calculations, we have observed the capability of interference in reducing the beam profile. Now, let us delve into a comprehensive discussion on the generation of ILS with exceptional performance. In LSFM, the uniformity of the illumination beam along the propagation axis and the beam width play crucial roles. Selecting appropriate parameters is essential to expand the FOV using the interference beam while preserving resolution and ensuring uniformity of light intensity. In this study, we first analyze the influence of interference beam parameters on the obtained results. Subsequently, we compare the characteristics of Gaussian beams, noninterference beams, and interference beams.

### 3.1 Influence of Distance Parameter α and Initial Phase Difference δ

To achieve optimal ILS effects, we systematically investigated the influence of interference parameters on the uniformity of light intensity and the beam cross-sectional profile. Initially, we focused on the uniform distribution of the interference beam along the propagation axis. We defined the uniformity of the interference beam by the intensity ratio R (α,δ), which represents the ratio of the intensity at the origin ($r_{s}=0,y_{s}=0$) to the intensity at the focal point ($r_{s}=0,y_{s}=\Delta$):

$$R(\alpha ,\delta )=I(r_{s}=0,y_{s}=0)/I(r_{s}=0,y_{s}=\Delta ).$$ (12)

**Fig. 2:**
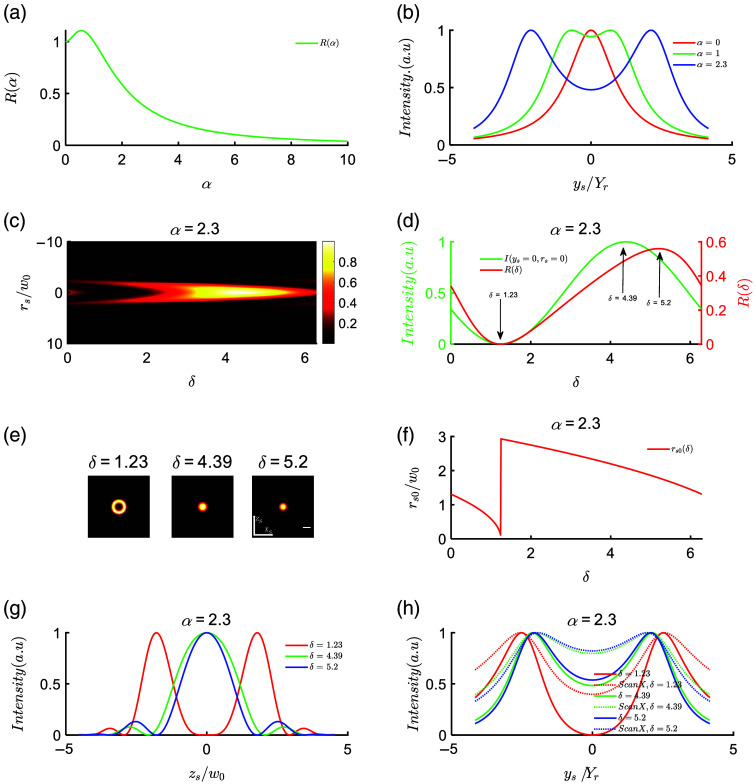
Influence of parameters α and initial phase difference δ. (a) Variation of the intensity ratio between $y_{s}=0$ and the focal point with respect to α. (b) Intensity curve along the $y_{s}$ axis. (c) The intensity variation at $y_{s}=0$ as a function of the radial coordinate $r_{s}$ and δ. In this plot, the radial coordinate $r_{s}$ is normalized by $w_{0}$, and the intensity is normalized by the maximum intensity. (d) The relationship curve between the intensity I ($r_{s}=0$, $y_{s}=0$) and δ. The green curve represents I ($r_{s}=0$, $y_{s}=0$) after being normalized by the maximum value, and the red curve represents the uniformity curve R(δ). (e) The variation of the beam cross-sectional intensity at different δ values, specifically δ=1.23, 4.39, and 5.2. (f) The relationship between the first zero of the intensity I $\left(r_{s},y_{s}=0\right)$ and the variation of δ. (g) Intensity $I(z_{s})$ at $y_{s}=0$ under different δ conditions, normalized to $w_{0}$ coordinates. (h) Variation of intensity along the propagation axis, solid line represents beam intensity, dashed line represents intensity after scanning the beam along the $x_{s}$ axis, normalized to Rayleigh length $Y_{r}$ coordinates. In (e), the scale is $2w_{0}$.

To determine the appropriate distance parameter α, we considered the conditions for interference enhancement, where cos(Φ) equals 1 in Eq. (9), corresponding to the origin ($r_{s}=0$, $y_{s}=0$). We plotted the variation of R with α, as shown in Fig. 2(a). When α is 0, the two beams overlap, resulting in R=1. As α increases, R initially rises, reaching its maximum value at α=0.56, before decreasing. For 0<α<1, the intensity at $y_{s}=0$ is greater than at $y_{s}=\Delta$. This indicates that for smaller α values, the intensity profile along the $y_{s}$ axis forms a single peak. With increasing α, the profile gradually splits into two peaks, resulting in a decrease in intensity between the peaks, as illustrated in Fig. 2(b). At α=2.3, which corresponds to a separation of 4.6 times the Rayleigh length between the two beams, the intensity at $y_{s}=0$, $r_{s}=0$ is 48.4% of the peak intensity, approximately half, as depicted by the blue solid line in Fig. 2(b).

To enhance the uniformity of light intensity by varying the initial phase (δ), we explore the impact of δ while keeping the distance parameter α constant. Figure 2(d) presents the intensity and uniformity at $y_{s}=0$, $r_{s}=0$ as a function of δ, with the intensity values normalized to their maximum. As δ increases from 0 to 2π, the intensity initially decreases (reaching a minimum at δ≈1.23), then gradually rises to its maximum (around δ≈4.39), followed by a decrease, exhibiting a cosine-shaped curve, as indicated by the green curve in Fig. 2(d). Correspondingly, the uniformity first decreases, then increases, and finally decreases again, with a minimum value of R(δ=1.23)=0 and a maximum value of R(δ=5.2)=56%. It is worth noting that the maximum values for both intensity and uniformity correspond to slightly shifted δ values. This discrepancy arises because changing δ not only affects the intensity at the origin but also impacts the intensity at other locations in space, as evident from the combined use of Eqs. (9) and (12). To further comprehend the influence of δ on the cross-sectional intensity, we plotted the radial light distribution at $y_{s}=0$ as a function of δ in Fig. 2(c). Around δ=1.23, destructive interference occurs at the origin, and as δ increases, the energy at the center ($r_{s}=0$) of the beam gradually strengthens. Near δ=4.39, interference enhancement occurs, resulting in maximum intensity. Figure 2(e) illustrates the intensity distributions at $y_{s}=0$ for δ values of 1.23, 4.39, and 5.2. The corresponding intensity profiles along the $z_{s}$ axis are shown in Fig. 2(g). At δ=1.23, destructive interference leads to the formation of a hollow beam [Fig. 2(e), red line in Fig. 2(g)]. Increasing δ causes the energy to converge toward the center, with maximum interference intensity occurring near δ=4.39. As δ continues to increase, the intensity at the origin starts to decrease, but the uniformity remains in an ascending phase [Fig. 2(d)]. The maximum uniformity is achieved at δ=5.2. Figure 2(h) provides a comparison of three scenarios: destructive interference (δ=1.23), interference enhancement (δ=4.39), and maximum uniformity (δ=5.2) for α=2.3. It displays the intensity distribution along the propagation axis ($y_{s}$) and the intensity distribution of the LS formed by scanning along the $x_{s}$ axis, with the intensity normalized to the maximum value. When destructive interference occurs, the intensity at the origin is zero, resulting in the poorest uniformity along the axial direction. At δ=5.2, both the uniformity of the beam intensity and the uniformity of the scanned LS (82.2%) are slightly superior to the scenario at δ=4.39 [indicated by the blue curve in Fig. 2(h)]. When the LS is formed by scanning along the $x_{s}$ axis, we observe that the intensity becomes more uniform along the $y_{s}$ axis, as depicted by the dashed line in Fig. 2(h). This is because in the scanning mode, the intensity along the $x_{s}$ axis is integrated, and thus the sidelobes contribute to improving the uniformity of the LS. In Fig. 2(h), we also observed a phenomenon where the maximum intensity varies with δ, similar to the focal shift phenomenon reported in previous studies.^31^

In addition to intensity uniformity, the size of the beam spot is equally important to consider. Using Eq. (10), we can plot the curve of the first zero point ($r_{s0}$) of the intensity distribution at the $y_{s}=0$ plane as a function of δ [Fig. 2(f)]. When δ is 0, $r_{s0}$ is $∼1.3w_{0}$. As δ gradually increases from 0 to 1.23, the intensity of the central lobe decreases, and the zero point moves closer to the center, resulting in a decrease in the first zero point as δ increases. Around δ=1.23, complete destructive interference occurs, causing the central lobe to disappear, and the first zero point becomes $r_{s0}=0$. As δ further increases, the central intensity remains nonzero, and the first zero point becomes the first zero point outside the central ring, leading to a sudden change in the curve as shown in Fig. 2(f). Subsequently, $r_{s0}$ decreases as δ increases, following a pattern consistent with Fig. 2(c). When δ is 5.2, $r_{s0}$ is $1.78w_{0}$. At this point, when the intensity of the main lobe drops to $1/e^{2}$ of its maximum, the corresponding $r_{s}$ is $1.386w_{0}$, which is smaller than the beam radius at the Rayleigh length of a Gaussian beam ($\surd 2w_{0}$).

Based on the analysis above, it is evident that interference beams exhibit side lobes, and there is an energy transfer between the main lobe and side lobes as the value of δ changes. Therefore, it is necessary to investigate the relationship between the main lobe, side lobes, and the value of δ in interference beams.

**Fig. 3:**
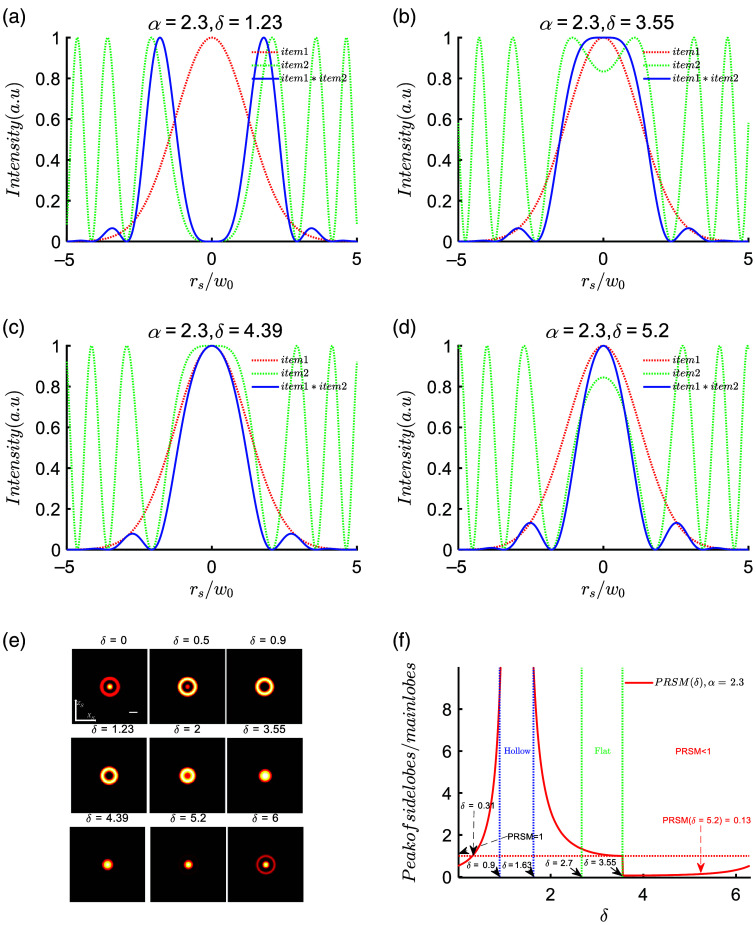
Modulation effect of δ on the beam profile. (a)–(d) The beam profiles at different δ values, where the red dashed line represents the first term of the interference intensity, the green dashed line represents the second term of the interference intensity, and the blue solid line represents the total interference intensity. The intensity values have been normalized. (e) The distribution of intensity at different δ values on the $z_{s}$, $x_{s}$ plane. (f) The peak intensity ratio between the side lobes and the central lobe. (PRSM: peak ratio of the first side lobe to the main lobe). The scale in (e) is $2w_{0}$.

According to Eq. (7), the interference intensity is the product of ($4I_{\Delta }$) and the cosine squared term. The first term is resembling the noninterfering beam at $y_{s}=0$, and the second term is representing the interference modulation. Figures 3(a)–3(d) illustrate the first term, second term, and interference intensity for α=2.3 at different δ values (1.23, 3.55, 4.39, and 5.2), with intensity normalization. When the two main peaks in the second term are widely separated, the interference intensity forms a hollow spot with a zero main lobe and the strongest side lobes [Fig. 3(a)]. As the two main peaks approach each other, the annular spot converges inward. Around δ=3.55, the interference intensity resembles a flat-top beam with side lobes peaking at ∼6.5% of the main lobe [Fig. 3(b)]. At δ=4.39, when the two peaks merge into one, the interference enhancement reaches its peak [Fig. 3(c)], with side lobes peaking at around 7.8% of the main lobe. Increasing δ further narrows the central peak of the second term, resulting in a thinner main lobe and a slight decrease in peak intensity while the side lobe intensity increases. At δ=5.2, the side lobes peak at ∼13% of the main lobe peak. Figure 3(e) presents the intensity distribution in the $y_{s}=0$ plane for various δ values, clearly demonstrating the evolution of the intensity profile with varying δ. Additionally, Fig. 3(f) shows the trend of the PRSM as a function of δ. When δ is <0.31, the main lobe is stronger than the side lobes. In the range of 0.31<δ<0.9, the side lobe energy increases rapidly. Within the range of 0.9<δ<1.63, the energy of the first side lobe significantly surpasses that of the central lobe, resulting in a hollow-like field. As δ ranges from 2.7 to 3.55, the PRSM approaches 1, resulting in a flat-top field. Importantly, when 3.55<δ<5.2, the main lobe predominates, with the first side lobe being very small (<0.13), and the second side lobe nearly absent [Figs. 3(c) and 3(d)]. This characteristic arises due to the exponential decay of side lobes as they are further away from the main lobe, distinguishing interference beams significantly from Bessel beam. As we all know, Bessel beam have multiple side lobes: the first side lobe typically carries around 16% of the main lobe’s peak intensity, followed by the second side lobe with ∼9%, and the third side lobe with about 8%… Each side lobe in a Bessel beam carries an equal amount of energy.^32^ In contrast, interference beams generally have only one side lobe, and the ratio of the side lobe’s peak intensity to the main lobe’s peak intensity can vary depending on the specific interference pattern and parameters.

In summary, we conducted a comprehensive study on the effects of distance parameter α and initial phase difference δ on the uniformity of light intensity, intensity distribution, and spot size. We observed that the uniformity of light intensity initially increases and then decreases as α increases. To expand the FOV, we selected two light beams separated by 4.6 Rayleigh lengths and investigated the influence of different δ values on the intensity distribution. Remarkably, when δ is set to 5.2, the intensity uniformity on the $r_{s}=0$ axis of the scanning plate reaches 82.2%. When δ is 5.2, the radius of the main lobe ($1/e^{2}$) is $1.386w_{0}$ ($<\surd 2w_{0}$). Additionally, we observed that the main lobe size ($r_{s0}$) of the spot decreases as δ increases, indicating that larger δ values contribute to smaller spot sizes. The modulation of interference terms resulted in variations in the beam profile with changing δ, including the emergence of hollow structures, flat distributions, or dominance of the main lobe. In comparison to Bessel beam, interference beams exhibit a significantly smaller number of side lobes due to exponential decay as the distance increases, highlighting a distinct advantage of interference beams.

### 3.2 Comparative Analysis of Three Beam Profiles: Uniformity of Intensity and Beam Width

**Fig. 4:**
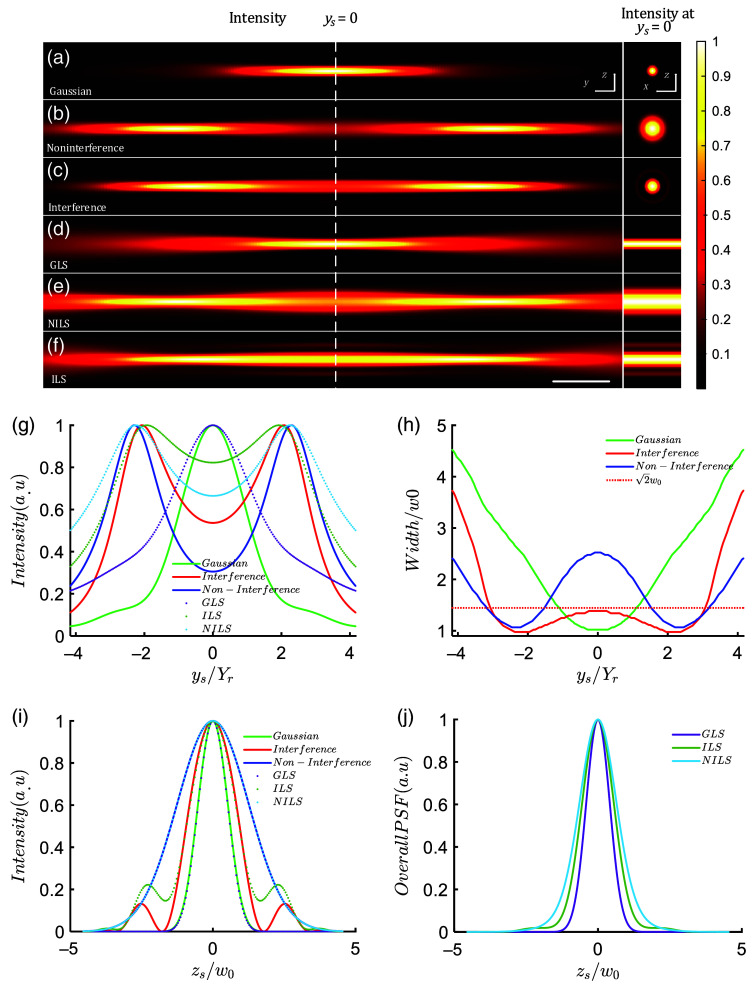
Comparison of three types of beams and LSs. (a)–(c) The intensity distributions of Gaussian beam, noninterference beam, and interference beam on the $y_{s}$, $z_{s}$ plane. (d)–(f) The intensity profiles of GLS, NILS, and ILS on the $y_{s}$, $z_{s}$ plane. The corresponding subplots on the right-hand side of (a)–(f) show the intensity distributions of the beams in the $y_{s}=0$ plane. (g) The intensity curves along the propagation axis ($y_{s}$) for the three types of beams and their corresponding LS. (h) The variation of beam width along the propagation axis for Gaussian beam, noninterference beam, and interference beam. (i) The intensity variation of $I(z_{s})$ at $y_{s}=0$ for the three types of beams and their corresponding LS. (j) The system point spread functions for the three types of LS. The parameters used are NA=0.16, α=2.3, λ=0.6328  nm, and ${\mathrm{NA}}_{d}=0.5$. The white dashed line indicates the focal plane where $y_{s}=0\;\mu m$. The scale in (a)–(f) is 20  μm.

To evaluate the performance of LSs formation using three different beams, we conducted simulations with the following parameters: illumination aperture NA=0.16, α=2.3, λ=0.6328  nm, δ=5.2; detection objective aperture ${\mathrm{NA}}_{d}=0.5$. Figures 4(a)–4(f) present the intensity distribution of the $y_{s}-z_{s}$ cross section and the intensity profile at $y_{s}=0$ for the three beams and their corresponding scanned LSs. The Gaussian beam focuses at the origin ($y_{s}=0$), whereas the two noninterfering beams and the interfering beam have their foci at a distance $\Delta =2.3Y_{r}$ from the origin ($y_{s}=0$). The Gaussian beam has the smallest spot size at the origin [Fig. 4(a)]. The noninterfering beam has a spot size of $∼2.5w_{0}$ at the origin, as shown in Fig. 4(b), the blue curve in Fig. 4(h), and its intensity rapidly decreases along the $y_{s}$ axis to 30.6% of the peak value [Fig. 4(g), the blue solid line]. However, when interference occurs, the beam size decreases [Fig. 4(c)], and the intensity at the origin increases to 53.7%, which is 1.75 times higher than the noninterfering case. The ILS achieves a uniformity of 82% [Fig. 4(g), dashed line], thus demonstrating better uniformity than the NILS. To assess the beam width, we define the beam width as the radial radius at which the intensity drops to $1/e^{2}$ of the peak value in the cross section. As shown in Fig. 4(h), the Gaussian beam has the smallest beam width, $w_{0}$, at the origin, and the beam width at a distance $Y_{r}$ from the origin is $\surd 2w_{0}$. Similar to the Gaussian beam, for both interference beams and noninterference beams, we can define the interval in which the beam width is less than the $\surd 2w_{0}$ as the effective length, representing the FOV of the LS. Therefore, the FOV for the GLS is $2Y_{r}$. If the two beams do not interfere, their intensities simply add up, resulting in a wider beam and an increased beam width of $2.5w_{0}$ at the origin. The effective length with a beam width smaller than $\surd 2w_{0}$ is only 3.12Yr [Fig. 4(h), solid blue line]. This means that noninterfering superposition leads to a degradation in beam quality because the two noninterfering beams rapidly diverge when moving away from their respective foci, and the diffused light overlaps with the other beam, resulting in an increased beam width. However, for interference beam, the beam width significantly decreases, and at $y_{s}=0$, the beam width is $∼1.386w_{0}$, smaller than $\surd 2w_{0}$ [Fig 4(h), solid red line]. The length with a beam width smaller than $\surd 2w_{0}$ is $∼5.88Y_{r}$ for interference beam. The intensity curves at $y_{s}=0$ are shown in Fig. 4(i), where the intensity profile of the interference beam is significantly smaller than that of the noninterference case due to the modulation by the cosine squared term. It should be noted that the interference beam exhibits side lobes; however, under the aforementioned parameters, the ratio of the first side lobe to the main peak is 0.13, smaller than $1/e^{2}$. Although the side lobes of the scanned beam formation lead to an undesired increased background intensity, with the background peak accounting for ∼0.2 of the main peak, the full width at half maximum (FWHM) of the ILS ($\mathrm{FWHM}=2w_{0}$) is reduced by 37.5% compared to the NILS ($\mathrm{FWHM}=3.2w_{0}$). The system point spread function of the LSFM is represented as the product of the detection point spread function and the illumination point spread function. As shown in Fig. 4(j), the point spread function of the ILS is smaller than that of the NILS case, and the interfering side lobes only result in a minor background intensity.

In summary, we systematically investigated the influence of the distance parameter α and the initial phase parameter δ on the interfering light field. Then under the condition of α=2.3, we optimized the initial phase difference to enhance the uniformity of the light intensity, resulting in an improved uniformity of 82.2% for the ILS. By utilizing the interference effect to modulate the beam profile, the thickness of the interfering LS was reduced by 37.5% compared to the NILS, effectively expanding the FOV of the LS to nearly three times that of the GLS.

## 4 Experiment

### 4.1 Sample Preparation

We have prepared two types of samples, namely a quantum dot fluorescent solution and fluorescent microspheres, in order to compare the performance of GLS, NILS, and ILS. The quantum dots used in our experiment are water-soluble cadmium-based quantum dots, with a concentration of 10  nm/ml and a photoluminescence full-width at half-maximum (PL/FWHM) of 671/40 nm. To observe the trajectory of the light beam, we introduce ∼10  μl of the quantum dot solution into a rectangular sample chamber filled with water (dimensions: 21.9  mm×63.3  mm×27  mm). As for the fluorescent microspheres, we utilize 0.2  μm silica fluorescent microspheres (632/680 nm). These microspheres are embedded in low-melting-point agarose at a ratio of 1:240 for the experiment.

### 4.2 Experimental Setup

To assess the performance of the ILS, we construct a custom experimental setup depicted in Fig. 1(b). We utilize a CW laser source (HNL100LB, 632.8 nm, 10 mw) as the excitation light, with its polarization aligned along the $x_{s}$ axis. The incident laser is split into red and blue light paths by a BS. In the red path, a retroreflector mirror adjusts the optical path length to control coherence. To regulate the relative focal distances of the two beams at the objective’s back focal plane (Olympus UPlanSApo 4×/0.16 NA), we employ an SLM (HOLOEYE, PLUTO-2) loaded with a defocus phase. The SLM is conjugated with the galvanometer mirrors and the objective’s entrance pupil. The other beam is directed into the common optical path through BS2. During the experiment, the blue light path is obstructed when imaging with the GLS. For ILS imaging, we adjust the mirror to ensure interference between the two beams. In the case of NILS imaging, a half-wave plate is inserted into the blue light path and rotated to render the light’s polarization perpendicular to the $x_{s}$ axis. Meanwhile, the optical path length of the red path is extended to prevent interference between the two beams. The sample is positioned on a high-precision motorized translation stage (NanoFaktur, SFS-D10600) capable of z axis movement. To capture fluorescence, we employ a water-immersion objective with a numerical aperture of 0.5 (Olympus UMPlanFl 20× Water Immersion, 0.5 NA) as the detection objective, and a tube lens (ETL = 200 mm) is positioned behind the detection objective. The detection objective lens is mounted on a movable translation stage, aligning the focal plane with the plane of LS. Fluorescence images are recorded by an sCMOS (ORCA-Flash BT C15440, Hamamatsu) camera at a rate of 50 frames per second. A filter (680 nm/FWHM 50 nm) is inserted in front of the camera to eliminate excitation light and stray light. The experiment involves imaging the trajectories of the optical paths and fluorescent microspheres.

## 5 Experimental Results

### 5.1 Experimental Results of Beam Fluorescence Trajectories

**Fig. 5:**
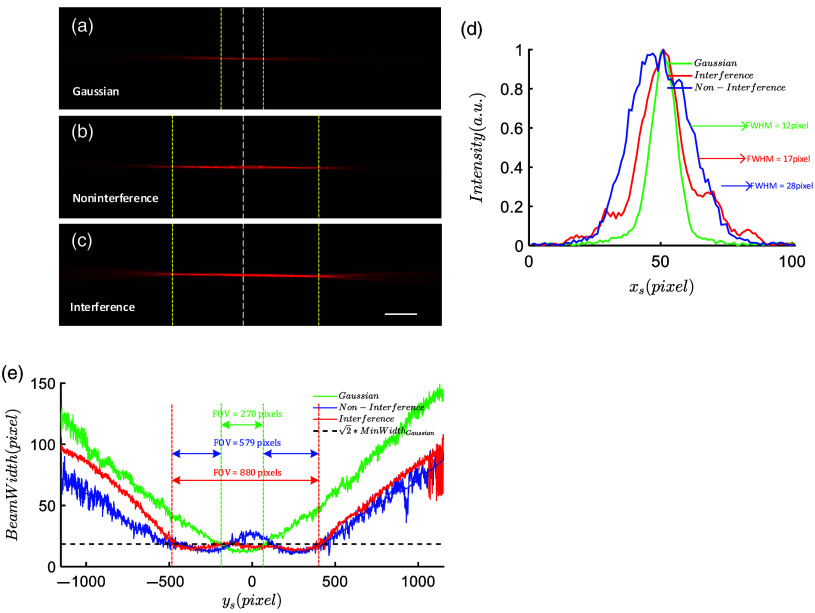
Experimental results of beam fluorescence trajectories in water-soluble quantum dot solution. (a) Gaussian beam fluorescence trajectory; (b) noninterfering beam fluorescence trajectory; (c) interfering beam fluorescence trajectory; and (d) beam fluorescence intensity distribution, corresponding to the white dashed line in the center ($y_{s}=0$) of the FOV. (e) The beam width variation curves along the propagation axis of Gaussian beams, noninterference beams, and interference beams. The black dashed line in the image represents the square root of 2 times the minimum beam waist width of the Gaussian beam. The yellow dashed lines represent the FOV boundaries on both sides, in (a)–(c), the yellow dashed line in (a) corresponds to the green dashed line in (e), whereas the yellow line in (b) and (c) corresponds to the red dashed line in (e). Scale: 63  μm.

The fluorescence trajectories of the beam in the quantum dot solution are shown in Fig. 5. For the Gaussian beam, the beam width is the narrowest only at the center ($y_{s}=0$) of the FOV, rapidly diverging away from the center, as illustrated in Fig. 5(a). If the spacing between two noninterfering beams focusing is greater than the Rayleigh length, the beam at the intermediate position of the focal point is noticeably thicker, as shown by the blue line in Figs. 5(b) and 5(d). When the two beams can interfere, the interference effect significantly reduces the beam width, as depicted in Figs. 5(c) and 5(d). Compared to noninterfering beams, the interference effect between the two beams results in a more uniform beam width in the FOV, as shown in Figs. 5(b) and 5(c). The interference beam width is it reduced by a factor of 1.647 compared to the noninterfering beam. For the interference beam, the width of the beam at the sides of the FOV are smaller, with the thickest part at the center, ∼1.416 times the minimum beam width of the Gaussian beam, as shown in Fig. 5(c). Compared to noninterference beams, interference beams exhibit the capability of reducing beam width and creating a flatter beam width curve. As shown in Fig. 5(e), for Gaussian beam, the effective FOV, where the beam width is less than the √2 times the Gaussian beam min waist width (Min ${\text{Width}}_{\text{Gaussian}}$), is ∼270  pixels (75.6  μm). In contrast, for noninterference beams, the effective FOV, where the beam width is less than √2 Min ${\text{Width}}_{\text{Gaussian}}$, is 579 pixels (162.1  μm). However, for interference beams, the effective FOV is 880 pixels (246.4  μm). Interference expands the FOV by ∼1.5 times compared to the noninterference situation. Therefore, compared to the NILS, the ILS has a smaller thickness and a longer effective length.

### 5.2 Experimental Results of Fluorescent Microsphere Imaging

**Fig. 6:**
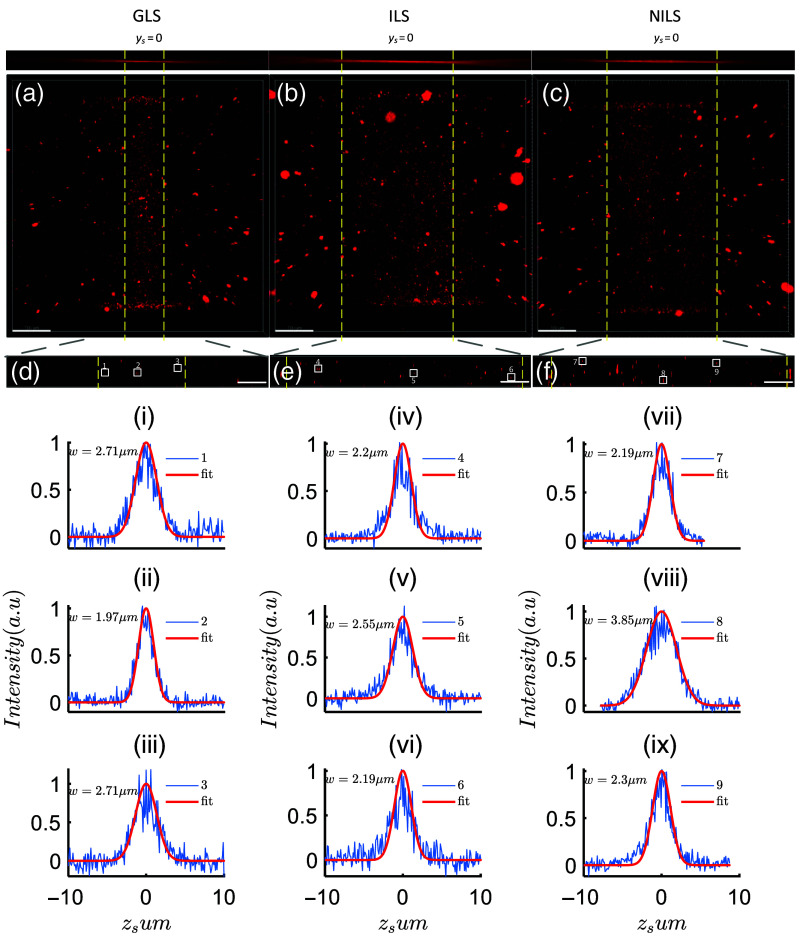
Three-dimensional imaging results of 0.2  μm fluorescent microspheres. (a) Microsphere imaging results illuminated by GLS. (b) Three-dimensional imaging results of microspheres illuminated by ILS. (c) Three-dimensional imaging results of microspheres illuminated by NILS. Panels (d)–(f) correspond to $y_{s}z_{s}$ cross sections in (a)–(c), respectively. (i)–(iii) The fluorescence intensity curves of microspheres numbered 1, 2, and 3 in (d) along the z direction. (iv)–(vi) The fluorescence intensity curves of microspheres numbered 4, 5, and 6 in (e) along the z direction. (vii)–(ix) The fluorescence intensity curves of microspheres numbered 7, 8, and 9 in (f) along the z direction. The scale in (a)–(c) is 100  μm, whereas the scale in (d)–(f) is 36  μm. The frame exposure time is 20 ms.

To further validate the performance of ILS, we conducted three-dimensional imaging of 0.2  μm silica fluorescent microspheres embedded in low-melting agarose blocks using GLS, NILS, and ILS as excitation LS. The imaging volume was 674×674×40  μm with a z axis step size of 0.1  μm. The imaging results are shown in Fig. 6, where the yellow dashed line represents the FOV boundary of the scanning LS.

First, for GLS illumination, the fluorescent microspheres in the center of the FOV appeared bright, whereas those farther from the center quickly became dim, as shown in Fig. 6(a). This observation is consistent with the intensity distribution characteristics of GLS along the propagation axis. In the case of ILS illumination, the region of the fluorescent microspheres illuminated in the FOV was noticeably larger, and the fluorescence intensity of the microspheres was more uniform, as shown in Fig. 6(b). However, for NILS, the fluorescence brightness of the microspheres near the center of the FOV ($y_{s}=0$) was significantly darker compared to the microspheres at the focal point of the scanning beam, as shown in Fig. 6(c). This is consistent with the results in Figs. 5(b) and 5(e), indicating that NILS have a larger thickness in the center, resulting in lower energy density.

To quantitatively analyze the axial resolution of the three types of LSs, we selected $y_{s}z_{s}$ cross sections from the three-dimensional volumes of the microspheres imaged by each LS [Figs. 6(d)–6(f)]. We chose microspheres close to $y_{s}=0$ and microspheres on both sides of the FOV, and then performed Gaussian fitting on their intensity along the $z_{s}$ axis to obtain their $z_{s}$ axis resolution [Figs. 6(i)–6(ix)].

First, taking the imaging results of fluorescent microspheres illuminated by GLS as an example, the $y_{s}z_{s}$ cross section is shown in Fig. 6(d). We selected microsphere 2 in the center of the FOV and microspheres 1 and 3 on both sides (close to the Rayleigh length). Then we plotted the intensity distribution curves of these three microspheres along the $z_{s}$ axis and performed Gaussian fitting, as shown in Figs. 6(i)–6(iii). The $1/e^{2}$ radius of microsphere 2 was 1.97  μm, microsphere 1 was 2.71  μm, and microsphere 3 was 2.71  μm. The calculated ratio of $z_{s}$ axis resolution differences was 2.71/1.97=1.376.

Next, taking the imaging results of fluorescent microspheres illuminated by ILS as an example, the $y_{s}z_{s}$ cross section is shown in Fig. 6(e). We selected three microspheres, microsphere 5 in the center of the FOV, and microspheres 4 and 6 on both sides, and plotted their intensity curves along the $z_{s}$ axis, as shown in Figs. 6(iv)–6(vi). The $1/e^{2}$ radii of microspheres 4, 5, and 6 were 2.2, 2.55, and 2.19  μm, respectively. The calculated ratio of Z axis resolution differences was 2.55/2.19=1.164.

Finally, taking the imaging results of fluorescent microspheres illuminated by NILS as an example, the $y_{s}z_{s}$ cross section is shown in Fig. 6(f). We selected three microspheres in the FOV and plotted their intensity distribution curves along the $z_{s}$ axis, as shown in Figs. 6(vii)–6(ix). The $z_{s}$ axis resolutions of microspheres 7, 8, and 9 were 2.19, 3.85, and 2.3  μm, respectively. The calculated ratio of $z_{s}$ axis resolution differences was 3.85/2.19=1.758.

Based on the data presented in Table 1, we are able to observe the axial resolution of three types of LSs along the $z_{s}$ axis. By combining our previous analysis with the findings from this table, we can draw the following conclusions: ILS exhibits a larger FOV compared to the other two LS types. Additionally, ILS demonstrates a more uniform axial resolution within the FOV. This implies that whether in the central or peripheral regions, we can expect consistent image quality and resolution when using ILS. Therefore, ILS demonstrates advantages in terms of both FOV and axial resolution.

**Table 1:** Axial resolution of three types of LSs in the left, center, and right FOV.

LS type	Left FOV (μm)	Center FOV (μm)	Right FOV (μm)	Axis resolution difference ratio
GLS	2.71	1.97	2.71	1.376
ILS	2.20	2.55	2.19	1.164
NILS	2.19	3.85	2.30	1.758

Note: The values in this table represent the axial resolution ($1/e^{2}$ radius) of the microspheres imaged using each type of LS in the corresponding FOV.

## 6 Discussion

Figure 5 demonstrates that the interference effect between two beams of light, compared to noninterfering light, reduces the beam width, and produces a more uniform beam profile. The experimental results with fluorescent microspheres in Fig. 6 provide evidence that the ILS exhibit a larger imaging FOV and a more uniform $z_{s}$ axis resolution within the FOV. The essence of expanding the FOV and achieving uniform resolution within the FOV lies in the cosine-squared modulation of the transverse intensity profile of the interference beams, with the radial coordinate squared. This modulation not only limits the beam divergence but also reduces the spot size, thereby increasing the energy density. Moreover, the improved uniformity of the intensity along the propagation axis of the ILS is attributed to the coherent enhancement of the beam.

Through theoretical derivations and simulations, we extensively discussed the sidelobe effects of the interfered beam. We discovered a specific range of δ values where the main lobe dominates, and the sidelobes exponentially decay with the square of the distance, resulting in the presence of almost a single sidelobe in the ILS. This is a significant difference from the numerous sidelobes observed in Bessel LSs.^33^^,^^34^ In the experimental trajectory of the interfered beam shown in Fig. 5(c), we indeed observed only one sidelobe, confirming our hypothesis. Additionally, we found that within certain ranges of δ, the sidelobes were significantly stronger than the central intensity, forming a so-called “hollow” light spot, which may find applications in particle trapping. Furthermore, through theoretical simulations, we discovered that by adjusting the initial phase difference δ alone, it is possible to reduce the spot size of the main lobe. Under appropriate conditions, subdiffraction-limited spots can be achieved, similar to previous research findings,^35^ which may have implications for super-resolution imaging.

In addition, we were surprised to find that the observed extent of FOV expansion in the experiment was higher than that in the simulated section. We speculate that this may be due to the presence of aberrations, such as spherical aberration during actual imaging. In the theoretical and simulated sections of the article, we only considered the ideal case without aberrations and assumed a sample space refractive index of 1. However, during actual imaging, there are media layers, such as the sample pool and water in the objective’s focal space, introducing spherical aberration and other aberrations. Studies have shown that spherical aberration indeed contributes to the expansion of the depth of focus.^36^ This suggests that the reduction in beam width by interference remains effective in the presence of aberrations, and spherical aberration may further expand the FOV. This finding warrants further research and exploration.

*Discussion on uniformity of axial resolution of LS*. Inconsistent thickness of LS can introduce variations in axial resolution within the FOV. The data presented in Table 1 demonstrate a decrease in the z axis resolution of microspheres near $y_{s}=0$ for both NILS and ILS. The NILS yields an axial resolution of ∼3.85  μm, with an axial resolution difference ratio of 1.758. Conversely, the ILS achieves an axial resolution of 2.55  μm, with an axial resolution difference ratio of 1.164. Although the axial resolution of ILS is slightly poorer than that of GLS near $y_{s}=0$, it is still slightly better than the axial resolution at the edge of GLS’s FOV. In contrast, the axial resolution of NILS near $y_{s}=0$ is significantly worse than the axial resolution of GLS. On the other hand, from the data provided in Table 1, we can observe that the NILS exhibits the poorest uniformity in terms of axial resolution. On the contrary, the ILS demonstrates a 1.51-fold enhancement in the axial resolution uniformity compared to the noninterference scenario (1.758/1.164). Furthermore, the ILS exhibits superior axial resolution uniformity compared to the GLS (1.376), owing to the beneficial interference effects introduced by the LSs.

*Comparison of ILS and nondiffraction LS*. As it is well known, Bessel beams exhibit numerous sidelobes, with the energy peak of the first sidelobe being ∼16%, the second sidelobe around 9%, and the third sidelobe about 8%^32^ … Due to the presence of these sidelobes, the background of Bessel LS exceeds 50%, thereby affecting the contrast and tomographic capabilities of imaging.^16^^–^^18^ In contrast, the sidelobe peak energy of ILS decays exponentially with the square of the distance [see Eqs. (7) and (8)], with higher-order sidelobes being almost negligible, leaving only a single sidelobe [Fig. 3(d)]. By adjusting the interference parameters (δ), it is possible to reduce the energy contribution of the primary sidelobe [Fig. 3(f)]. Under the condition of δ=5.2, this sidelobe contributes ∼25% to the background of the ILS [Fig. 4(i) and 5(d)]. Therefore, it can be speculated that ILS may possess higher imaging contrast, assuming the same LS thickness. Airy LS are known to have not only numerous sidelobes but also a curved beam shape, which leads to image distortion. Consequently, additional calibration and deconvolution postprocessing are required for Airy LS, which is not conducive to real-time imaging.^13^^–^^15^ Furthermore, although lattice LS offers excellent tomographic capabilities and resolution, their generation requires complex optical field modulation techniques and precise mask templates for amplitude filtering, resulting in low transmission efficiency.^19^^,^^20^ Thus simplifying the generation process of lattice LS and improving their transmission efficiency remains a challenging research problem. In comparison to Bessel LS, Airy LS, and lattice LS, ILS possess several advantages. It has fewer sidelobes, thereby providing higher contrast and tomographic capabilities. Additionally, ILS enables real-time imaging without the need for additional calibration and deconvolution, unlike Airy LS. Compared to lattice LS, ILS does not require complex optical field modulation techniques or precise mask templates, resulting in improved transmission efficiency. The generation of ILS is simple and efficient, requiring only two interfering light beams to produce the ILS.

Another important topic for discussion is the impact of light scattering in biological tissues on ILS. Most biological tissues are complex structures composed of components with varying concentrations and refractive indices.^37^^,^^38^ These structures span from 10 nm to several tens of micrometers. According to Mie scattering theory and Rayleigh scattering theory, this scattering may lead to changes in polarization, degradation of coherence, and the formation of random speckles, ultimately affecting the imaging quality of the ILS. Research has shown that refractive index mismatch is a significant factor causing light scattering in biological tissues.^37^ Fortunately, tissue clearing technology can enhance the uniformity of tissue refractive index and reduce heterogenous absorbers, such as pigments, to minimize light scattering and absorption within the tissue. Through tissue clearing techniques, transparency has been achieved in tissues and organs at various scales,^39^^–^^42^ and even at the level of individual organisms.^43^^–^^46^ By utilizing the coherence and interference properties of lasers and optimizing the shape of light patterns through techniques, such as optical field modulation, combined with tissue transparency techniques, we have enabled imaging of biological tissues at millimeter to centimeter scales.^47^^–^^52^ Therefore, it can be inferred that by combining tissue transparency techniques with ILS microscopy, significant reduction in light scattering and absorption can be achieved while preserving the characteristics of ILS, thereby enabling three-dimensional imaging of various transparent biological samples.

Through the utilization of a BS to separate the light into two beams and subsequent coupling with BS to generate an ILS, the optical path difference in this configuration is susceptible to environmental influences. Another potential approach involves the use of diffractive elements like SLM to generate ILS, which could simplify the optical setup and enhance system stability. By employing SLM, phase modulation can be directly introduced into beam, leading to the creation of multiple LS. In addition, leveraging the dynamic phase adjustment capability of SLM, the characteristics of the ILS can be dynamically tailored as per requirements. Furthermore, SLMs offer improved stability to the optical system. With real-time phase adjustment, precise control over the optical path difference can be achieved, minimizing the impact of environmental factors on the ILS. In the future, employing diffractive elements, such as spatial light modulators to generate ILS will be an effective approach, simplifying the optical setup, enhancing system stability, and offering flexibility in optimizing the interference field.

## 7 Conclusion

We have compared the performance of GLS, NILS, and ILS in terms of imaging FOV and z axis resolution through simulations and fluorescence microsphere experiments. The results demonstrate that the ILS provides a larger FOV compared to the NILS and GLS and exhibits a more uniform axial resolution within the FOV. This confirms that the interference effect of multiple LS can optimize the distribution of light intensity, thereby expanding the FOV and achieving a more uniform axial resolution in LSFM.

We have analyzed and discussed the reasons behind the improvement in imaging quality by ILS, attributing it primarily to the modulation of light intensity through the cosine squared term. Based on this observation, we speculate that appropriate selection of interference parameters can contribute to the generation of subdiffraction-limited spots, potentially enabling super-resolution imaging.

In summary, our research emphasizes the importance of ILS in LSFM. By harnessing the interference effect, we can expand the FOV and enhance imaging quality, offering potential opportunities for super-resolution imaging. Further research will contribute to a deeper understanding of the advantages and application potential of ILS, providing guidance and inspiration for the development of LSFM.

## Data Availability

Data underlying the results presented in this paper are not publicly available at this time but may be obtained from the authors upon reasonable request.

## References

[1] Corsetti S. Gunn-Moore F. Dholakia K., “Light sheet fluorescence microscopy for neuroscience,” J. Neurosci. Methods 319, 16–27 (2019). JNMEDT 0165-0270 10.1016/j.jneumeth.2018.07.011 30048674

[r2] Hillman E. M. C. et al., “Light-sheet microscopy in neuroscience,” Annu. Rev. Neurosci. 42, 295–313 (2019). ARNSD5 0147-006X 10.1146/annurev-neuro-070918-050357 31283896 PMC6800245

[3] Mano T. et al., “Whole-brain analysis of cells and circuits by tissue clearing and light-sheet microscopy,” J Neurosci 38, 9330–9337 (2018). JNNUEF 10.1523/JNEUROSCI.1677-18.2018 30381424 PMC6706004

[4] Huisken J. Stainier D. Y. R., “Selective plane illumination microscopy techniques in developmental biology,” Development 136, 1963–1975 (2009). 10.1242/dev.022426 19465594 PMC2685720

[5] Keller P. J. et al., “Digital scanned laser light-sheet fluorescence microscopy (DSLM) of zebrafish and drosophila embryonic development,” Cold Spring Harbor Protoc. 2011, 1235–1243 (2011). 10.1101/pdb.prot065839 21969622

[6] Ritter J. G. Veith R. Veenendaal A. Siebrasse J. P. Kubitscheck U., “Light sheet microscopy for single molecule tracking in living tissue,” PLoS One 5, e11639 (2010). POLNCL 1932-6203 10.1371/journal.pone.0011639 20668517 PMC2909143

[7] de Medeiros G. et al., “Multiscale light-sheet organoid imaging framework,” Nat. Commun. 13, 4864 (2022). NCAOBW 2041-1723 10.1038/s41467-022-32465-z 35982061 PMC9388485

[8] Huisken J. et al., “Optical sectioning deep inside live embryos by selective plane illumination microscopy,” Science 305, 1007–1009 (2004). SCIEAS 0036-8075 10.1126/science.1100035 15310904

[r9] Keller P. J. et al., “Reconstruction of zebrafish early embryonic development by scanned light sheet microscopy,” Science 322, 1065–1069 (2008). SCIEAS 0036-8075 10.1126/science.1162493 18845710

[10] Becker K. et al., “Ultramicroscopy: 3D reconstruction of large microscopical specimens,” J. Biophotonics 1, 36–42 (2008). 10.1002/jbio.200710011 19343633

[11] Olarte O. E. et al., “Light-sheet microscopy: a tutorial,” Adv. Opt. Photonics 10, 111–179 (2018). AOPAC7 1943-8206 10.1364/AOP.10.000111

[12] Durnin J. Miceli J. J. Eberly J. H., “Diffraction-free beams,” Phys. Rev. Lett. 58, 1499–1501 (1987). PRLTAO 0031-9007 10.1103/PhysRevLett.58.1499 10034453

[13] Liu P. et al., “Airy beam assisted NIR-II light-sheet microscopy,” Nano Today 47, 101628 (2022). NTAOCG 1748-0132 10.1016/j.nantod.2022.101628

[r14] Yang Z. et al., “A compact Airy beam light sheet microscope with a tilted cylindrical lens,” Biomed. Opt. Express 5, 3434–3442 (2014). BOEICL 2156-7085 10.1364/BOE.5.003434 25360362 PMC4206314

[15] Vettenburg T. et al., “Light-sheet microscopy using an Airy beam,” Nat. Methods 11, 541–544 (2014).1548-7091 10.1038/nmeth.2922 24705473

[16] Fahrbach F. O. et al., “Self-reconstructing sectioned Bessel beams offer submicron optical sectioning for large fields of view in light-sheet microscopy,” Opt. Express 21, 11425–11440 (2013). OPEXFF 1094-4087 10.1364/OE.21.011425 23669999

[r17] Meinert T. Rohrbach A., “Light-sheet microscopy with length-adaptive Bessel beams,” Biomed. Opt. Express 10, 670–681 (2019). BOEICL 2156-7085 10.1364/BOE.10.000670 30800507 PMC6377868

[18] Luna-Palacios Y. Y. et al., “Multicolor light-sheet microscopy for a large field of view imaging: a comparative study between Bessel and Gaussian light-sheets configurations,” J. Biophotonics 15, e202100359 (2022). 10.1002/jbio.202100359 35184408

[19] Tsai Y.-C. et al., “Rapid high resolution 3D imaging of expanded biological specimens with lattice light sheet microscopy,” Methods 174, 11–19 (2020). MTHDE9 1046-2023 10.1016/j.ymeth.2019.04.006 30978505

[20] Chen B.-C. et al., “Lattice light-sheet microscopy: imaging molecules to embryos at high spatiotemporal resolution,” Science 346(6208), 1257998 (2014). SCIEAS 0036-8075 10.1126/science.1257998 25342811 PMC4336192

[21] Xiong B. et al., “Improving axial resolution of Bessel beam light-sheet fluorescence microscopy by photobleaching imprinting,” Opt. Express 28, 9464–9476 (2020). OPEXFF 1094-4087 10.1364/OE.388808 32225553

[22] Chen Y. et al., “A versatile tiling light sheet microscope for imaging of cleared tissues,” Cell Rep. 33, 108349 (2020). 10.1016/j.celrep.2020.108349 33147464

[23] Gao L., “Extend the field of view of selective plan illumination microscopy by tiling the excitation light sheet,” Opt. Express 23, 6102–6111 (2015). OPEXFF 1094-4087 10.1364/OE.23.006102 25836834

[24] Hedde P. N. Gratton E., “Selective plane illumination microscopy with a light sheet of uniform thickness formed by an electrically tunable lens,” Microsc. Res. Tech. 81, 924–928 (2016). MRTEEO 1059-910X 10.1002/jemt.22707 27338568 PMC5479743

[r25] Kim B. et al., “Open-top axially swept light-sheet microscopy,” Biomed. Opt. Express 12, 2328–2338 (2021). BOEICL 2156-7085 10.1364/BOE.419030 33996232 PMC8086456

[26] Liu Y. Rollins A. M. Jenkins M. W., “CompassLSM: axially swept light-sheet microscopy made simple,” Biomed. Opt. Express 12, 6571–6589 (2021). BOEICL 2156-7085 10.1364/BOE.440292 34745757 PMC8547981

[27] Li H. et al., “Axially overlapped multi-focus light sheet with enlarged field of view,” Appl. Phys. Lett. 118, 223701 (2021). 10.1063/5.0049013

[28] Liu C. et al., “Extended field of view of light-sheet fluorescence microscopy by scanning multiple focus-shifted Gaussian beam arrays,” Opt. Express 29, 6158–6168 (2021). OPEXFF 1094-4087 10.1364/OE.418707 33726142

[29] Richards B. Wolf E., “Electromagnetic diffraction in optical systems, II. Structure of the image field in an aplanatic system,” Proc. R. Soc. Lond. A 253, 358–379 (1959). PRLAAZ 1364-5021 10.1098/rspa.1959.0200

[30] Alda J., “Laser and Gaussian beam propagation and transformation,” Encyclopedia Opt. Eng. 999, 1013 (2003).

[31] Li Y., “Focal shift and focal switch in dual-focus systems,” J. Opt. Soc. Am. A 14, 1297–1304 (1997). JOAOD6 0740-3232 10.1364/JOSAA.14.001297

[32] Mori S., “Side lobe suppression of a Bessel beam for high aspect ratio laser processing,” Precis. Eng. 39, 79–85 (2015). PREGDL 0141-6359 10.1016/j.precisioneng.2014.07.008

[33] Fahrbach F. O. et al., “Light-sheet microscopy in thick media using scanned Bessel beams and two-photon fluorescence excitation,” Opt. Express 21(11), 13824–13839 (2013). OPEXFF 1094-4087 10.1364/OE.21.013824 23736637

[34] Deng S. et al., “Subtraction method via phase mask enables contrast enhancement in scanned Bessel light-sheet microscopy,” J. Opt. Soc. Am. A 37(1), 84–88 (2020). 10.1364/JOSAA.37.000084 32118884

[35] Yun M. et al., “Tunable transverse superresolution with phase-only pupil filters,” J. Opt. A: Pure and Appl. Opt. 8, 1027 (2006). CJOEE3 1671-7694 10.1088/1464-4258/8/12/001

[36] Tomer R. et al., “SPED light sheet microscopy: fast mapping of biological system structure and function,” Cell 163(7), 1796–1806 (2015). CELLB5 0092-8674 10.1016/j.cell.2015.11.061 26687363 PMC4775738

[37] Johnsen S. Widder E. A., “The physical basis of transparency in biological tissue: ultrastructure and the minimization of light scattering,” J. Theor. Biol. 199(2), 181–198 (1999). JTBIAP 0022-5193 10.1006/jtbi.1999.0948 10395813

[38] Tuchin V. V., “Tissue optics and photonics: light-tissue interaction,” J. Biomed. Photonics Eng. 1(2), 98–134 (2015). 10.18287/JBPE-2015-1-2-98

[39] Chung K. et al., “Structural and molecular interrogation of intact biological systems,” Nature 497, 332–337 (2013). 10.1038/nature12107 23575631 PMC4092167

[r40] Susaki E. A. et al., “Whole-brain imaging with single-cell resolution using chemical cocktails and computational analysis,” Cell 157(3), 726–739 (2014). CELLB5 0092-8674 10.1016/j.cell.2014.03.042 24746791

[r41] Ertürk A. et al., “Three-dimensional imaging of solvent-cleared organs using 3DISCO,” Nat. Protoc. 7(11), 1983–1995 (2012).1754-2189 10.1038/nprot.2012.119 23060243

[42] Stefaniuk M. et al., “Light-sheet microscopy imaging of a whole cleared rat brain with Thy1-GFP transgene,” Sci. Rep. 6(1), 28209 (2016). 10.1038/srep28209 27312902 PMC4911560

[43] Gómez-Gaviro M. V. et al., “Optimized CUBIC protocol for three-dimensional imaging of chicken embryos at single-cell resolution,” Development 144(11), 2092–2097 (2017). 10.1242/dev.145805 28432219

[r44] Belle M. et al., “Tridimensional visualization and analysis of early human development,” Cell 169(1), 161–173.e12 (2017). CELLB5 0092-8674 10.1016/j.cell.2017.03.008 28340341

[r45] Pan C. et al., “Shrinkage-mediated imaging of entire organs and organisms using uDISCO,” Nat. Methods 13(10), 859–867 (2016).1548-7091 10.1038/nmeth.3964 27548807

[46] Tainaka K. et al., “Whole-body imaging with single-cell resolution by tissue decolorization,” Cell 159(4), 911–924 (2014). CELLB5 0092-8674 10.1016/j.cell.2014.10.034 25417165

[47] Chen B.-C. et al., “Lattice light-sheet microscopy: imaging molecules to embryos at high spatiotemporal resolution,” Science 346(6208), 1257998 (2014). SCIEAS 0036-8075 10.1126/science.1257998 25342811 PMC4336192

[r48] Gao R. et al., “Cortical column and whole-brain imaging with molecular contrast and nanoscale resolution,” Science 363(6424), eaau8302 (2019). SCIEAS 0036-8075 10.1126/science.aau8302 30655415 PMC6481610

[r49] Chen B. et al., “Resolution doubling in light-sheet microscopy via oblique plane structured illumination,” Nat. Methods 19, 1419–1426 (2022).1548-7091 10.1038/s41592-022-01635-8 36280718 PMC10182454

[r50] Wang D. et al., “Tiling light sheet selective plane illumination microscopy using discontinuous light sheets,” Opt. Express 27, 34472–34483 (2019) OPEXFF 1094-4087 10.1364/OE.27.034472 31878494

[r51] Fang C. et al., “Wide-view Bessel light-sheet fluorescence microscopy for high-resolution, isotropic neuron imaging,” in Conf. Lasers and Electro-Opt. Pac. Rim (CLEO-PR), IEEE (2018). 10.1364/CLEOPR.2018.Tu3K.4

[52] Takanezawa S. Saitou T. Imamura T., “Wide field light-sheet microscopy with lens-axicon controlled two-photon Bessel beam illumination,” Nat. Commun. 12(1), 2979 (2021). NCAOBW 2041-1723 10.1038/s41467-021-23249-y 34016994 PMC8137944

